# Case Report: rapid exacerbation of autoimmune hemolysis and severe immune - mediated thrombocytopenia induced by piperacillin – tazobactam

**DOI:** 10.3389/fmed.2026.1844022

**Published:** 2026-06-17

**Authors:** Juan Liu, Zhongliang Shi, He Zhang, Mingfang Qian

**Affiliations:** 1 Department of Nephrology, Hangzhou Linping Hospital of Traditional Chinese Medicine, Hangzhou, Zhejiang, China; 2 Department of Critical Care Medicine, Tongde Hospital of Zhejiang Province, Hangzhou, Zhejiang, China; 3 Zhejiang Academy of Traditional Chinese Medicine, Hangzhou, Zhejiang, China

**Keywords:** adverse drug reaction, drug-induced immune hemolytic anemia, drug-induced thrombocytopenia, immune - mediated, piperacillin-tazobactam

## Abstract

**Background:**

Piperacillin-tazobactam (PIP-TAZ) is characterized by its broad antimicrobial spectrum, strong antibacterial activity, and low toxicity, playing a vital role in the treatment of infectious diseases. However, with its widespread use, reports of drug-induced immune hemolytic anemia (DIIHA) and drug-induced thrombocytopenia (DITP) have begun to emerge. Due to the relatively rare clinical incidence of these conditions, clinicians often have an inadequate understanding and lack corresponding management experience. Fortunately, we encountered a case in clinical practice that we quickly identified and successfully treated.

**Case presentation:**

An 83-year-old Chinese female patient was treated with PIP-TAZ for pneumonia. On the fourth day of treatment, she exhibited pronounced symptoms of jaundice, accompanied by elevated total bilirubin levels, severe hemolytic anemia and thrombocytopenia. Upon investigating the underlying cause, we found that the concentration of PIP-TAZ in her blood was significantly higher than the normal therapeutic range, and PIP-TAZ-dependent antibodies were detected in her serum. We ultimately diagnosed her with DIIHA combined with DITP. Following prompt recognition and management, the patient made a full recovery and was subsequently discharged.

**Conclusion:**

This article presents a case of acute immune hemolytic anemia and immune thrombocytopenia induced by PIP-TAZ. Effective treatment strategies include the prompt identification and discontinuation of the offending drug, blood transfusions, intravenous immunoglobulin (IVIG), corticosteroid therapy, and careful monitoring of vital signs.

## 1 Introduction

Drug-induced immune hemolytic anemia (DIIHA) is a rare but severe adverse reaction, with an incidence of approximately one in a million (1, 2). PIP-TAZ is one of the common antibiotics that can induce DIIHA, following cefotetan and ceftriaxone in terms of frequency (3, 4). DIIHA is primarily caused by the induction of drug-specific antibodies by the drug itself or its metabolites. These antibodies primarily target an autoantigen present on the red blood cell (RBC) membrane, resulting in the direct destruction of RBCs (5). Drug-induced thrombocytopenia (DITP) is a rare and frequently overlooked cause of thrombocytopenia. The mechanism underlying thrombocytopenia induced by PIP-TAZ remains unclear; however, studies suggest that it may be mediated by immune mechanisms (6). Despite the immune mechanism being the mainstream explanation, the specific structural fragments of the drug that elicit this immunogenic response have yet to be identified. The structure-activity relationship responsible for generating the immune response remains unclear. This uncertainty presents a significant clinical challenge; due to the structural similarities among β-lactam antibiotics, there is a potential for cross-reactivity.

Currently, the diagnosis of DIIHA primarily relies on correlational analyses between the patient’s clinical history and manifestations of hemolytic anemia. This is supplemented by positive results from the Direct Antiglobulin Test (DAT) for anti-IgG and/or anti-C3d. Additionally, it includes the detection of drug-induced antibodies, which encompasses both drug-dependent and drug-independent antibodies, while systematically excluding other potential factors that may contribute to these symptoms. The clinical diagnosis of DITP similarly relies on a clear temporal association between drug exposure and the onset of thrombocytopenia. The gold standard for confirming DITP is the demonstration of drug-dependent anti-platelet antibodies in the patient’s serum. However, it is crucial to note that negative results from anti-platelet antibody tests do not exclude the diagnosis of DITP associated with PIP-TAZ (7).

This article presents a case report of a patient who experienced a rapid exacerbation of DIIHA and severe DITP induced by PIP-TAZ. The aim of this report is to enhance clinicians’ understanding of the clinical characteristics and management of PIP-TAZ-induced immune hemolytic anemia and immune thrombocytopenia. The study received approval from the Ethics Committee of the Zhejiang Academy of Traditional Chinese Medicine (Hangzhou, Zhejiang, China) under approval number 2026-044 and is drafted in accordance with the CARE (Case Report) guidelines (8).

## 2 Case presentation

An 83-year-old Chinese female patient has been hospitalized in the rehabilitation department for 2 years due to the sequelae of heat stroke, primarily characterized by neurological deficits, particularly cognitive and memory impairments. These impairments have resulted in her inability to perform daily activities independently. During this hospitalization, the patient experienced fatigue without any identifiable triggers. At that time, her vital signs were recorded as follows: temperature: 36.7 °C, pulse: 72 beats per minute, respiration rate: 18 breaths per minute, and blood pressure: 130/63 mmHg. Laboratory tests revealed a significant increase in inflammatory markers (Table 1): the white blood cell (WBC) count was 10.0 × 10^∧^9/L, C-reactive protein (CRP) was 199.98 mg/L, and procalcitonin (PCT) was 47.578 ng/mL. Other laboratory data included RBCs at 2.75 × 10^∧^12/L, hemoglobin (Hb) at 86 g/L, and platelet (PLT) count at 178 × 10^∧^9/L. Liver and kidney function, as well as coagulation function, were generally within normal ranges. No significant abnormalities were observed during the physical examination, which included assessments of the skin, chest, abdomen, and nervous system.

**TABLE 1:** Laboratory data on admission in this subject.

Variable	Result	Reference range	Variable	Result	Reference range
Peripheral blood			Blood biochemistry		
WBC (10E9/L)	10.0	3.5–9.5	T.Bil (μmol/L)	9.1	0.0–21.0
Neutrophil (%)	72.8	40.0–75.0	D.Bil (μmol/L)	2.6	0.0–7.5
Lymphocyte (%)	20.8	20.0–50.0	I.Bil (μmol/L)	6.5	1.7–19.0
Monocyte (%)	5.9	3.0–10.0	Albumin(g/L)	31.2	40.0–55.0
EOS (%)	0.3	0.4–8.0	Globulin (g/L)	39.8	20.0–40.0
Baso (%)	0.2	0.0–1.0	ALT (U/L)	20	7–40
RBC (10E12/L)	2.75	3.80–5.10	AST (U/L)	34	15–35
Hemoglobin (g/L)	86	115–150	ALP (U/L)	99	30–120
Hematocrit (%)	26.0	35.0–45.0	LDH (U/L)	165	120–250
Platelets (10E9/L)	178	125–350	GGT (U/L)	39	7–45
Coagulation function	Result	Reference range	Glucose(mmol/L)	6.86	3.89–6.11
PT(s)	12.0	9.8–12.5	Urea (mmol/L)	5.9	3.1–8.8
INR	1.05	0.84–1.10	Creatinine (μmol/L)	82	40–83
APTT (s)	35.7	25.0–31.3	Uric acid (μmol/L)	193	140–340
TT (s)	16.0	15.0–20.5	Potassium (mmol/L)	3.66	3.50–5.30
Fibrinogen (g/L)	7.14	1.90–3.90	Sodium (mmol/L)	137.0	137.0–147.0
D-dimer (mg/L)	1.06	0.00–0.50	Chloride (mmol/L)	100.0	99.0–110.0
Urinary test	Result	Reference range	Total Ca (mmol/L)	2.06	2.11–2.52
Urinary pH	7.0	5.4–8.4	TC (mmol/L)	3.53	3.10–5.70
Urinary protein	2+	–	Cholinesterase (U/L)	5870	4000–13000
Urinary sugar	–	–	Amylase (U/L)	61	35–135
Urinary ketone	–	–	CRP (mg/L)	199.98	0–6.00
Urinary blood	–	–	Procalcitonin (ng/mL)	47.578	<0.065
Urobilirubin	–	–	NT–proBNP (pg/mL)	2488	<125

ALT, alanine aminotransferase; AST, aspartate aminotransferase; ALP, alkaline phosphatase; ATPP, activated partial prothrombin time; Baso, basophilic granulocyte; Ca, calcium; CRP, C-reactive protein; D.Bi, direct bilirubin; EOS, eosinophilic granulocyte; GGT, gamma glutamyl transpeptidase; I.Bil, indirect bilirubin; INR, international normalized ratio; LDH, lactate dehydrogenase; NT-proBNP, N-terminal pro-brain natriuretic peptide; PT, prothrombin time; RBC, red blood cells; TC, Total cholesterol; TT, thrombin time; T.Bil, total bilirubin; WBC, white blood cell.

Additionally, the N-terminal pro b-type natriuretic peptide (NT-proBNP), a biomarker for heart failure, was significantly elevated at 2488 pg/mL. A computed tomography scan of the chest revealed the presence of scattered exudates, atelectasis, and inflammation in both lungs (Supplementary Figure 1). Consequently, the attending physician initiated antibiotic treatment for pneumonia with intravenous PIP-TAZ at a dosage of 4.5 g every 8 h, while also prescribing oral furosemide to alleviate cardiac load. On the second and third days, the patient primarily experienced fatigue, with no other prominent clinical symptoms observed. However, on the fourth day, the patient presented with jaundice and multiple ecchymoses on the skin. Despite these symptoms, the physical examination of the abdomen—including the liver, spleen, and lymph nodes—revealed no significant abnormalities, prompting the attending physician to reassess the relevant laboratory indicators. The patient’s inflammatory markers exhibited significant deterioration, with a WBC count of 10.7 × 10^∧^9/L, CRP level at 223.2 mg/L, and PCT exceeding 99.250 ng/mL. Additionally, abnormalities in coagulation function were noted, including a prothrombin time of 17.0 s, an international normalized ratio of 1.52, an activated partial thromboplastin time of 34.4 s, a fibrinogen level of 4.29 g/L, and a D-dimer level exceeding 30.00 mg/L. Furthermore, the PLT count dramatically decreased from 178 × 10^∧^9/L to 1 × 10^∧^9/L. The patient also developed significant hemolytic anemia, as evidenced by the following laboratory results: RBC count of 1.17 × 10^∧^12/L, Hb level of 38 g/L, hematocrit of 10.8%, total bilirubin of 54.7 μmol/L, direct bilirubin of 22.8 μmol/L, indirect bilirubin of 31.9 μmol/L, lactate dehydrogenase of 944 U/L, creatinine of 400 μmol/L, and urea of 29.6 mmol/L.

Due to the patient’s critical condition, they were transferred to the Intensive Care Unit (ICU) for further treatment. In the ICU, we investigated the potential causes of hemolytic anemia, with the following relevant data: the DAT returned a positive result, while no other autoantibodies, including those associated with systemic lupus erythematosus, were detected. Furthermore, we have ruled out the possibility of bleeding from the gastrointestinal tract and thoracoabdominal cavities. The tests for gastric juice and stool occult blood returned negative results, while ultrasound examinations of the thoracic and abdominal cavities showed no significant abnormalities. Antigen tests for Streptococcus pneumoniae and Legionella returned negative results. Additionally, tests for Parvovirus B19, Cytomegalovirus, Hepatitis B and C viruses, as well as Influenza A and B viruses, and Mycoplasma pneumoniae antigens and/or antibodies were also negative. A bone marrow puncture was conducted, which revealed no abnormalities, thereby excluding the possibility of hematologic diseases. Monitoring of drug concentrations in the blood revealed that the concentration of Piperacillin was 499 μg/ml. The Naranjo scale yielded a score of 8 points, indicates probable adverse drug reaction. Additionally, we identified IgG-dependent piperacillin-related antibodies in the plasma, with a titer of 68, after culturing with a piperacillin solution and RBCs at 37 °C. No abnormal red blood cell antibodies or tazobactam-dependent antibodies were detected. A multidisciplinary discussion concluded that the drug may have induced DIIHA.

**FIGURE 1:**
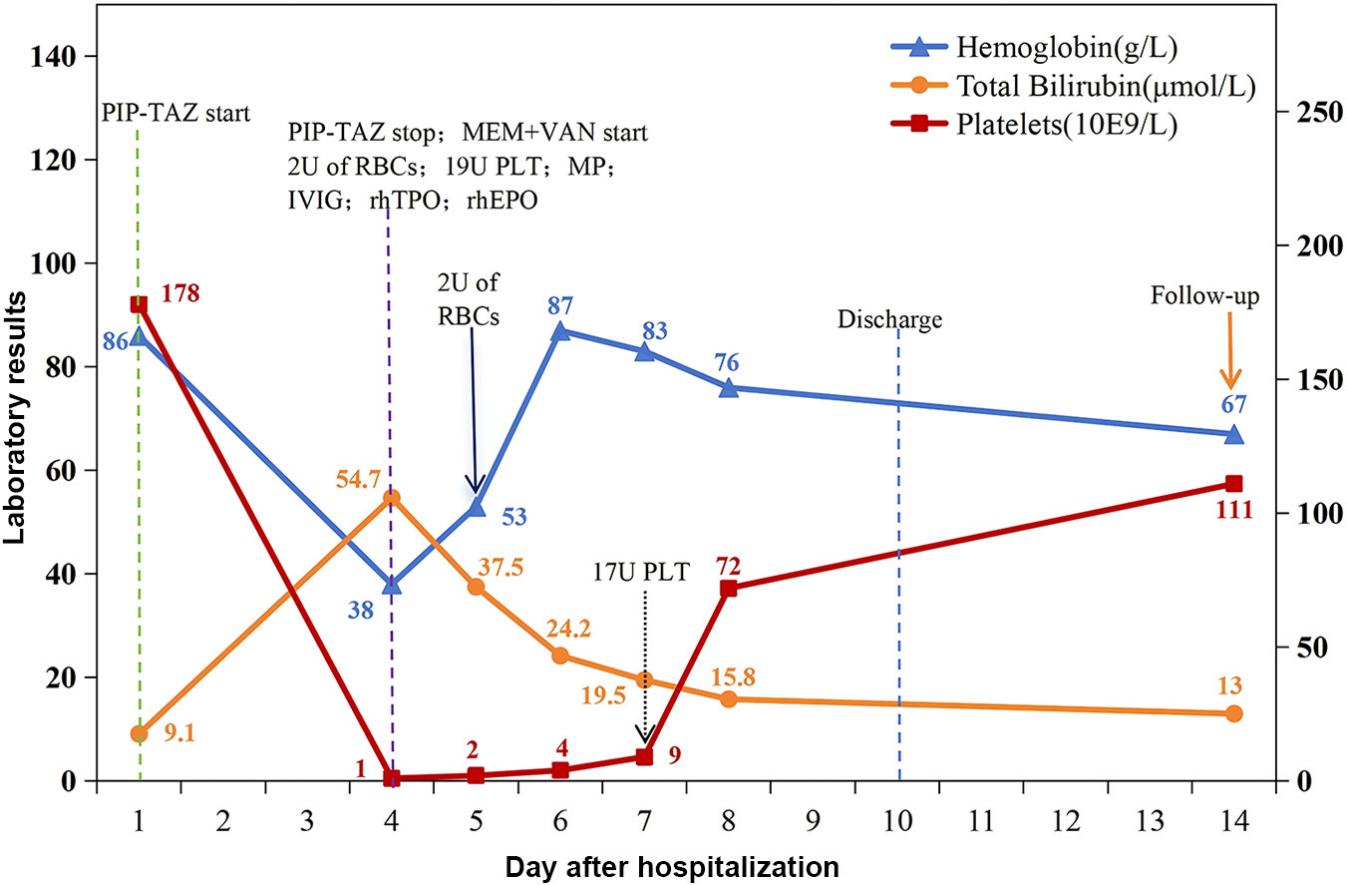
The changes in hemoglobin, platelets, and total bilirubin. IVIG, intravenous immunoglobulin; MEM, meropenem; MP, methylprednisolone; PIP-TAZ, Piperacillin-tazobactam; PLT, platelet; RBCs, Red blood cells; rhTPO, recombinant human thrombopoietin; rhEPO, recombinant human erythropoietin; VAN, vancomycin.

In the treatment protocol, we promptly discontinued PIP-TAZ and transitioned to an antibiotic regimen consisting of meropenem 1 g IV every 8 h and vancomycin 0.5 g IV every 12 h. This change was necessitated by a significant elevation in the patient’s infection indicators, which required empirical coverage for both gram-positive and gram-negative bacteria. Concurrently, the patient received 2 units of RBCs and 19 units of platelets. Additionally, an injection of recombinant human thrombopoietin (rhTPO) was administered at a dose of 15,000 units once daily to enhance platelet counts, along with recombinant human erythropoietin (rhEPO) at 10,000 units three times per week to address anemia. Additionally, a combination therapy of methylprednisolone at 40 mg every 12 h and immunoglobulin G at 20 g once daily (0.4 g/kg) was administered as a 3-day pulse treatment. On the fifth day, the patient’s hemoglobin level had increased to 53 g/L; however, the platelet count remained low at 2 × 10^∧^9/L. The RBC transfusion proved effective, prompting the administration of an additional 2 units of RBCs. By the sixth day, the hemoglobin level had risen to the patient’s baseline of 87 g/L. Due to the persistent low platelet count, we administered 17 units of platelets on the seventh day, when the platelet count remained low at 9 × 10^∧^9/L. By the eighth day, the platelet count had risen to 72 × 10^∧^9/L. Following a series of treatments, the patient’s clinical symptoms and laboratory indicators demonstrated significant improvement (Figure 1 and Table 2). Ultimately, the patient was successfully discharged after 10 days of treatment in the ICU. We advised the patient to exercise caution regarding future use of PIP-TAZ, as immune hemolytic reactions may exhibit immunological memory. This can lead to a more rapid increase in the production of antibodies against drug-red blood cell complexes upon re-exposure to the same medication, potentially resulting in more rapid and severe hemolytic reactions (9, 10). It is noteworthy that the DAT remained positive for 10 days after the cessation of the drug. Fifteen days following the patient’s discharge, a follow-up was conducted, and the direct Coombs test returned negative. In the subsequent year, we followed up with the patients’ families, indicating that no long-term sequelae occurred. Furthermore, there was no further administration of PIP-TAZ, nor was there any recurrence of hemolysis.

**TABLE 2:** Inflammation markers, hemolytic markers and coagulation function after admission.

Inflammation markers	Day 1	Day 4	Day 5	Day 6	Day 7	Day 8	Day 14	Reference range
WBC (10E9/L)	10.0	10.7	6.7	8.3	8.1	8.8	10.0	3.5–9.5
Neut (%)	72.8	84.5	84.0	89.7	84.2	79.3	75.4	40.0–75.0
CRP (mg/L)	199.98	223.2	134.55	88.74	52.22	30.18	28.63	0–6.00
PCT (ng/mL)	47.578	99.250	-	77.373	40.970	17.871	0.265	<0.065
**Hemolytic markers**	**Day 1**	**Day 4**	**Day 5**	**Day 6**	**Day 7**	**Day 8**	**Day 14**	**Reference range**
RBC (10E12/L)	2.75	1.17	1.73	2.89	2.72	2.54	2.16	3.80–5.10
Hb (g/L)	86	38	53	87	83	76	67	115–150
T.Bil (μmol/L)	9.1	54.7	37.5	24.2	19.5	15.8	13.0	0.0–21.0
D.Bil (μmol/L)	2.6	22.8	19.9	9.5	6.0	5.0	3.1	0.0–7.5
I.Bil (μmol/L)	6.5	31.9	17.6	14.7	13.5	10.8	9.9	1.7–19.0
LDH (U/L)	165	944	-	398	-	341	240	120–250
DAT		+					+	
**Coagulation function**	**Day 1**	**Day 4**	**Day 5**	**Day 6**	**Day 7**	**Day 8**	**Day 14**	**Reference range**
PT (s)	12.0	17.0	16.2	13.7	12.7	12.3	12.3	9.8–12.5
INR	1.05	1.52	1.44	1.21	1.11	1.07	1.07	0.84–1.10
APTT (s)	35.7	34.4	36.7	36.9	35.3	30.5	32.8	25.0–31.3
TT (s)	16.0	16.9	17.2	18.1	19.7	18.8	15.4	15.0–20.5
FIB (g/L)	7.14	4.29	2.70	1.60	1.06	1.42	2.84	1.90–3.90
D-d (mg/L)	1.06	30.00	30.00	30.00	30.00	25.52	4.05	0.00–0.50
Platelets(10E9/L)	178	1	2	4	9	72	111	125–350

ATPP, activated partial prothrombin time; CRP, C-reactive protein; DAT, direct antiglobulin test; D.Bi, direct bilirubin; D-d, D-dimer; FIB, fibrinogen; Hb, hemoglobin; I.Bil, indirect bilirubin; INR, international normalized ratio; LDH, lactate dehydrogenase; Neut, neutrophil; PCT, procalcitonin; PT, prothrombin time; RBC, red blood cell; T.Bil, total bilirubin; TT, thrombin time; WBC, white blood cell.

## 3 Discussion

This article presents a successful case of treating a patient with DIIHA and DITP caused by PIP-TAZ. The effective treatments implemented included the immediate cessation of the medication, blood transfusions, IVIG, and corticosteroid therapy, among others.

Drug-induced immune hemolytic anemia is a complex condition primarily mediated by immune mechanisms. The academic community generally agrees that the mechanisms underlying DIIHA can be categorized into two core areas: drug-induced antibodies and non-immune protein adsorption (NIPA) (11–13). Drug-induced antibodies are the most critical immunological factors in the occurrence of DIIHA, and their modes of action can be further subdivided into four types. The first mode, termed ‘drug only’ or the drug adsorption mechanism, involves the direct binding of the drug to the red blood cell membrane. The second mode, referred to as ‘drug + membrane,’ describes the typical immune complex mechanism in which the drug binds to red blood cell membrane proteins, resulting in the formation of a drug-membrane complex. The third mode, ‘membrane predominant,’ indicates that antibodies, such as IgG warm autoantibodies, can directly bind to RBCs independent of the drug. The fourth mode integrates elements from the preceding three, suggesting that antibodies induced by the same drug may concurrently exhibit characteristics of drug adsorption, immune complexes, and autoantibody-like properties. Unlike drug-induced antibodies, NIPA represents a fundamentally different mechanism. Its primary characteristic is the modification of the RBC membrane by the drug, which facilitates the non-immunologic and nonspecific adsorption of various plasma proteins, including immunoglobulins such as IgM and IgG, complement system proteins, and albumin onto the surface of RBCs. Notably, nearly all cases of significant anemia in DIIHA are attributed to drug-induced antibodies (12, 14). In contrast, the NIPA mechanism results solely in a positive DAT and is characterized by slow and subtle hemolysis (15, 16).

Drug-induced antibodies can be further categorized into drug-dependent antibodies and drug-independent antibodies. The mechanism of action for non-drug-dependent antibodies primarily involves the induction of autoantibodies through drug-induced immune responses, Notably, even after the drug is discontinued, these autoantibodies may persist in the body. In contrast, drug-dependent antibodies react exclusively with RBCs in the presence of the drug (17). Most cases of apparent DIIHA are attributed to drug-dependent antibodies, which manifest as immune hemolytic anemia. There is a significant correlation between the timing of drug administration and the onset of hemolysis; typically, after the discontinuation of the drug, hemolysis often alleviates and gradually ceases. In this case, the patient’s hemolytic symptoms were rapidly alleviated following the prompt discontinuation of piperacillin. Consequently, we propose that these symptoms may be attributed to drug-dependent antibodies that induce hemolysis.

In cases of DIIHA caused by PIP-TAZ, nearly all reports indicate a positive result for anti-IgG antibodies in the DAT (1, 3, 4, 18–20). Most cases present with acute intravascular hemolysis. However, it is important to note that approximately 5%–10% of cases may yield false-negative results in the DAT (21). The onset of hemolysis following the initiation of PIP-TAZ can occur within a range of 2 h to 16 days, with hemoglobin levels decreasing by 34 to 78 g/L (22). The serological test results for this patient indicated a positive DAT for anti-IgG, clinically manifesting as acute hemolytic anemia. The hemoglobin level decreased significantly from 86 g/L to 38 g/L, closely correlating with the timing of PIP-TAZ administration. Concurrently, we monitored drug concentrations and found that the piperacillin concentration in the blood was 499 μg/ml, while the tazobactam concentration was 53.8 μg/ml. It is noteworthy that in DIIHA, this reaction is mediated by the immune system and is not solely dependent on drug concentration; however, elevated drug concentrations do increase the likelihood of immune complex formation. Additionally, the serum sample was collected during a period of severe hemolysis in the patient. The *in vitro* hemolytic process can lead to the release of drugs from within RBCs into the bloodstream, potentially resulting in an overestimation of drug concentration in the serum. Furthermore, due to the patient’s acute renal failure, the renal excretion of the drug and its metabolites is impeded, which may also result in elevated blood drug concentrations. While it is not possible to entirely exclude the potential for hemolysis associated with sepsis or pneumonia (23), it is clear that DIIHA is the predominant cause in this case.

Drug-induced thrombocytopenia is a rare yet often overlooked etiology of thrombocytopenia. The exact mechanism by which PIP-TAZ induces thrombocytopenia is still unclear; however, theories propose that it may be mediated by an immune response (24). Drug-dependent antibodies may target platelets, contributing to thrombocytopenia. The thrombocytopenia associated with PIP-TAZ primarily involves two distinct mechanisms. The first mechanism is a pseudo-reduction, wherein the core process involves drug-dependent antibody-mediated *in vitro* platelet aggregation. This process results in the irreversible aggregation of platelets within the blood collection tube, forming clumps that are not recognized by blood cell analyzers. Consequently, these aggregates are excluded from the platelet count channel, leading to a falsely significant decrease in the reported platelet count. Importantly, the platelets in the body are not genuinely consumed or destroyed (25). The other mechanism is a true reduction, which signifies a genuine, *in vivo* immune-mediated destruction of platelets, wherein PIP-TAZ functions as a hapten, prompting the immune system to generate specific IgG antibodies (26, 27).

Previous studies have reported that PIP-TAZ primarily induces immune hemolysis of RBCs, with less pronounced effects on platelets. However, in our patient, the platelet count dramatically decreased from 178 × 10^∧^9/L to 1 × 10^∧^9/L. Additionally, after consulting with the laboratory, we performed blood smear microscopy on the original blood specimen and did not observe significant platelet aggregation. This study has a limitation in that we did not conduct further tests to determine the presence of platelet antibodies against PIP-TAZ. However, the presence of RBC antibodies suggests that platelet antibodies related to the drug may also be present, as the transfusion of platelets proved to be completely ineffective. Clinically, this was evidenced by a significant decrease in platelet counts among patients exposed to PIP-TAZ, accompanied by notable petechiae and ecchymoses on the skin. We suspect that DITP may have occurred. It is noteworthy that the patient exhibited elevated inflammatory markers at that time, indicating a potential risk of sepsis. Given the observed changes in coagulation function on day 4 and an ISTH score of 8, the possibility of overt disseminated intravascular coagulation (DIC) must be considered. Consequently, in addition to the drug-induced immune-mediated destruction of platelets, factors associated with sepsis and diffuse intravascular coagulation should also be taken into account.

In cases of immune-mediated platelet destruction, repeated platelet transfusions are often ineffective due to the rapid destruction of transfused platelets by antibodies. Given the patient’s multiple ecchymoses and significant bleeding risk, we administered 19 units of platelets, which were completely ineffective. This observation indirectly suggests that the patient’s thrombocytopenia may be associated with immune-mediated platelet destruction. Following treatment with IVIG and corticosteroids, we administered an additional 17 units of platelets, resulting in an increase from 9 × 10^∧^9/L to 72 × 10^∧^9/L. It is important to note that this increase may also be related to the control of the patient’s DIC and sepsis during this period.

For DIIHA and DITP, in addition to symptomatic treatments such as discontinuing medication and administering transfusions, most case reports suggest that glucocorticoids at a dosage of 80 mg/day administered intravenously, IVIG at a dosage of 0.4–1 g/kg/day (28–34), rhEPO to promote erythropoiesis (5000–10000 IU, administered subcutaneously three times a week), and rhTPO at 300 IU/kg/day to enhance platelet production have demonstrated certain effectiveness (35). The treatment was ultimately very successful, resulting in a rapid recovery and discharge of the patient. It is noteworthy that the patient’s hemoglobin level continuously decreased from day 6 (87 g/L) to day 14 (67 g/L), which is significantly lower than the level at admission (Table 2). We believe this may be related to the patient’s occurrence of occult blood in the stool, which was positive at + + on day 8 and persisted until day 18, when the stool occult blood test returned negative. Upon re-examination on day 30, the patient’s hemoglobin had risen to 91 g/L.

It is important to emphasize that life-threatening hemolytic anemia and thrombocytopenia caused by a single drug are extremely rare and inadequately reported. Most case studies focus solely on the manifestations of hemolytic anemia, neglecting the complications associated with thrombocytopenia. Mechanistically, these two pathological processes are distinctly different; however, they may share a common pathogenic basis: the drug acts as a hapten, binding to blood cell membrane proteins and triggering the production of drug-dependent antibodies. When these antibodies simultaneously recognize the drug-protein complexes on the surfaces of RBCs and platelets, a reduction in both types of blood cells may occur. Therefore, when using PIP-TAZ, it is essential to monitor not only for hemolysis but also for a potential decrease in platelet count. Regardless of which type of blood cell reduction is involved, discontinuation of the drug remains the primary measure. It is noteworthy that this study is based solely on case reports, which cannot establish causality and may be subject to limitations, such as potential confounding factors. Future prospective studies and additional case reports are necessary to clarify the immunological cross-reactivity patterns of this drug across different blood cell lineages, thereby enhancing its safety monitoring strategies.

## 4 Conclusion

During treatment with PIP-TAZ, it is essential to maintain vigilance for DIIHA and DITP. Timely serological testing is crucial for diagnosis. If DIIHA is suspected, a DAT should be performed immediately. If the IgG test result is positive, drug-dependent antibodies must be confirmed using drug-coated RBCs in a specialized laboratory to establish the diagnosis. Similarly, detecting platelet antibodies against PIP-TAZ is a key method for diagnosing DITP, and this testing method is analogous to RBC testing. Furthermore, discontinuation of PIP-TAZ is the most effective strategy to prevent serious complications associated with its administration. We present a case of DIIHA and DITP resulting from the administration of PIP-TAZ. Fortunately, we promptly identified the issue and discontinued the use of this medication immediately. Furthermore, we implemented effective therapeutic measures, including blood transfusions, immunoglobulin therapy, corticosteroids, and erythropoietin, all of which contributed to a successful outcome.

## Data Availability

The original contributions presented in this study are included in this article/Supplementary material, further inquiries can be directed to the corresponding author.

## Supplementary material

Supplementary Figure 1 The results of the computed tomography scan of the chest.
