# Supplementary figures and images for: Case Report: rapid exacerbation of autoimmune hemolysis and severe immune - mediated thrombocytopenia induced by piperacillin – tazobactam
Source: Front Med (Lausanne). 2026 Jun 17;13:1844022. doi: 10.3389/fmed.2026.1844022 (PMC13318588; doi:10.3389/fmed.2026.1844022)

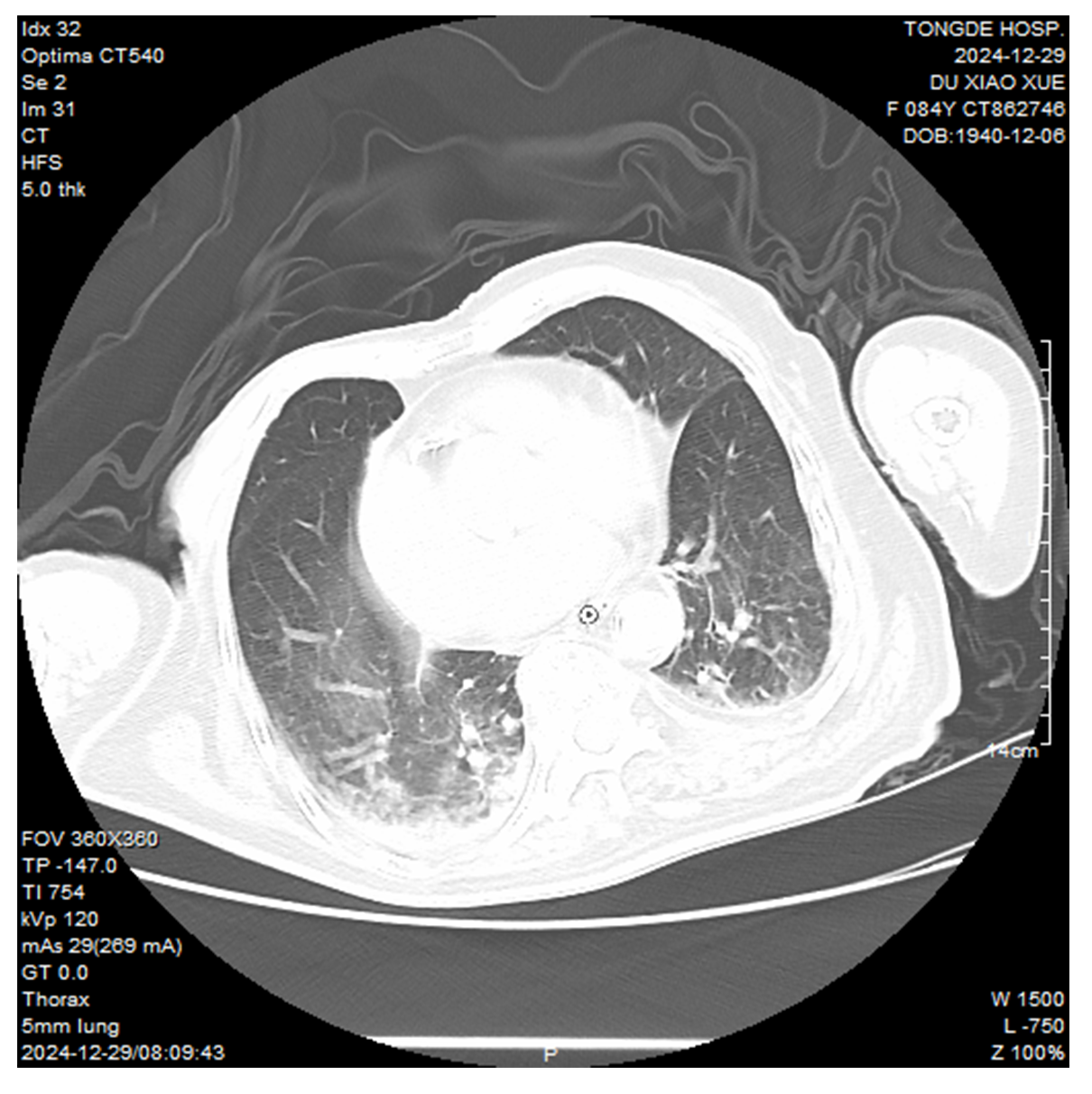

Supplement: Supplementary Figure 1 — The results of the computed tomography scan of the chest. [file Image_1.tif]
